# The Stress Index as a Predictor of Mortality in Patients with Isolated Moderate to Severe Traumatic Brain Injury

**DOI:** 10.3390/diagnostics14121244

**Published:** 2024-06-13

**Authors:** Ching-Ya Huang, Cheng-Shyuan Rau, Chun-Ying Huang, Wei-Ti Su, Shiun-Yuan Hsu, Ching-Hua Hsieh

**Affiliations:** 1 Kaohsiung Chang Gung Memorial Hospital, Chang Gung University College of Medicine, Kaohsiung 83301, Taiwan; b101106030@tmu.edu.tw; 2 Department of Neurosurgery, Kaohsiung Chang Gung Memorial Hospital, Chang Gung University College of Medicine, Kaohsiung 83301, Taiwan; ersh2127@adm.cgmh.org.tw; 3 Department of Trauma Surgery, Kaohsiung Chang Gung Memorial Hospital, Chang Gung University College of Medicine, Kaohsiung 83301, Taiwan; junyinhaung@yahoo.com.tw (C.-Y.H.); s101132@adm.cgmh.org.tw (W.-T.S.); ah.lucy@hotmail.com (S.-Y.H.)

**Keywords:** trauma, traumatic brain injury (TBI), stress index (SI), risk factor, mortality

## Abstract

Background: The Stress Index (SI), calculated as the ratio of blood glucose to serum potassium levels, is a promising prognostic marker in various acute care settings. This study aimed to evaluate the utility of the SI for predicting mortality in patients with isolated moderate-to-severe traumatic brain injury (TBI). Methods: This retrospective cohort study included adult trauma patients (aged ≥ 20 years) with isolated moderate to severe TBI (Abbreviated Injury Scale ≥ 3 for only head region) treated from 2009–2022. The SI was computed from the initial glucose and potassium levels upon arrival at the emergency department. Logistic regression models were used to assess the association between the SI and mortality after adjusting for relevant covariates. The most effective threshold value of the SI for predicting mortality was identified using receiver operating characteristic (ROC) analysis. Results: Among the 4357 patients with isolated moderate and severe TBI, 463 (10.6%) died. Deceased patients had a significantly higher SI (61.7 vs. 44.1, *p* < 0.001). In multivariate analysis, higher SI independently predicted greater mortality risk (odds ratio (OR) 6.70, 95% confidence interval (CI) 1.66–26.99, *p* = 0.007). The optimal SI cutoff for predicting mortality was 48.50 (sensitivity 62.0%, specificity 71.4%, area under the curve 0.724). Patients with SI ≥ 48.5 had nearly two-fold higher adjusted mortality odds compared to those below the threshold (adjusted OR 1.94, 95% CI 1.51–2.50, *p* < 0.001). Conclusions: SI is a useful predictor of mortality in patients with isolated moderate-to-severe TBI. Incorporating SI with standard clinical assessments could enhance risk stratification and management approaches for this patient population.

## 1. Introduction

Potassium is essential for regulating the electrical potential difference across cell membranes, which is critical for nerve and muscle functions, including those in the heart [1]. Trauma can disrupt potassium balance, leading to hyperkalemia or hypokalemia, both of which can have serious consequences for heart and muscle function as well as fluid and acid-base balance [2,3,4], which play a crucial role in the outcomes of patients with trauma and significantly influence their recovery. Hypokalemia is common in patients with trauma, occurring in 50–68% of cases, and is often linked to acute stress responses in the body [2]. This condition, characterized by a rapid decrease in potassium levels within hours of trauma, can indicate severe injury, and is associated with longer hospital stays and an increased need for intensive care [3]. Conversely, hyperkalemia presents serious risks, especially during the early resuscitation phase, and is associated with an increased cardiac risk [5]. Studies have shown that patients with TBI are particularly vulnerable to potassium imbalances, necessitating more aggressive potassium supplementation and careful monitoring [6]. Overall, maintaining optimal potassium levels through vigilant monitoring and timely intervention is essential for improving the clinical outcomes of patients with trauma. On the other hand, the blood glucose levels are tightly regulated under normal circumstances, but trauma can induce a strong stress response that alters hormone levels and affects glucose homeostasis. The secretion of stress hormones, such as adrenaline and cortisol, can result in hyperglycemia, which has the potential to inhibit immunological function, heighten susceptibility to infection, and impede wound healing [7,8,9,10]. Conversely, hypoglycemia can cause confusion, dizziness, and even coma, potentially resulting in severe damage to the brain and other vital organs. Both potassium and glucose play crucial roles in maintaining physiological homeostasis, and their imbalance can have profound effects on patient health.

The Stress Index (SI), or glucose–potassium ratio, is defined as the ratio of blood glucose to serum potassium levels and has received substantial attention as a valuable predictive tool in the management of patients under critical care [11,12,13,14,15,16,17]. The clinical relevance of the SI lies in its ability to capture the intricate interplay between hyperglycemia, a hallmark of the body’s stress response, and shifts in potassium levels, which signal cellular damage or systemic metabolic disturbances [7,8,9,10]. These physiological alterations serve as critical markers of disease severity and can guide urgent medical interventions, making the SI a potentially life-saving asset in time-sensitive scenarios. Numerous studies across various emergency care settings have demonstrated the predictive efficacy of the SI [11,12,13,14,15,16,17]. In cases involving massive transfusions, damage control operations, or severe trauma, elevated SI levels have been linked to unfavorable outcomes, underscoring the utility of the SI in forecasting the necessity for these critical interventions [17]. This simple yet powerful index provides a rapid and objective measure of the physiological stress response of the body, reflecting the acute metabolic changes that occur during acute medical conditions. Supporting evidence exists regarding the prognostic value of SI in critical care settings [18].

The ability to rapidly identify patients requiring urgent care is paramount in the initial hours following trauma when swift decision-making can significantly influence patient survival and long-term prognosis. Prognostic scoring systems, such as Acute Physiology and Chronic Health Evaluation II (APACHE II) [19], new Simplified Acute Physiology Score (SAPS II) [20], Mortality Probability Models II (MPM II) [21], and Sepsis-related Organ Failure Assessment (SOFA) [22], have been extensively researched for the purpose of categorizing trauma patients with severe illness. Nevertheless, these systems sometimes require time and resources to collect the data and demand a significant number of variables or specific fundamental attributes. The intricacies of patients with traumatic brain injury (TBI) require a rapid prognostic approach that can identify even small changes in patient condition and offer real-time insights that are crucial for making dynamic decisions and achieving optimal management. Research has highlighted the relevance of the SI in predicting mortality in patients with subarachnoid hemorrhage and ischemic stroke, where alterations in glucose to potassium levels correspond with patient outcomes [12,13,15]. This broader applicability underscores the versatility of the SI as a prognostic marker across a spectrum of acute care settings, both traumatic and non-traumatic. In acute care settings, particularly for patients with TBI, rapid and accurate assessment tools are critical for guiding therapeutic interventions to improve patient outcomes. Recent studies have also explored the potential of the SI as a prognostic factor in patients sustaining severe subarachnoid hemorrhage [16]. These investigations revealed a significant association between higher SI values and poorer outcomes, suggesting its potential utility as a biomarker for predicting prognosis and guiding initial management decisions, such as the necessity for surgical intervention at the time of hospital admission.

Given the complex physiological changes associated with profound TBI, including disruptions in metabolic processes and electrolyte imbalances, SI may provide vital information regarding a patient’s overall physiological stress resulting from trauma. The SI may aid in the formulation of more precise and efficient management approaches, specifically for patients with moderate-to-severe TBI, by virtue of its ease and simplicity of computation. This, in turn, could contribute to enhanced long-term recovery outcomes and increased survival rates. Therefore, this study aimed to assess the utility of the SI as a prognostic tool for mortality in patients with moderate-to-severe TBI.

## 2. Materials and Methods

### 2.1. Patient Enrollment and Study Design

Prior to commencing the research, the Institutional Review Board (IRB) of Chang Gung Memorial Hospital approved the procedure (approval number 202400311B0). Due to the retrospective nature of the study, patient permission was not required. This study employed a retrospective cohort design, examining data from adult patients with trauma aged 20 or above with isolated moderate to severe traumatic brain injury (TBI), as indicated by an Abbreviated Injury Scale (AIS) score of ≥3 only in the head area [23,24,25,26]. Medical data were obtained from the Trauma Registry System at the hospital between 1 January 2009 and 31 December 2022. The exclusion criteria encompassed individuals with certain types of injuries such as burns, hanging injuries, and drowning, as well as those with missing data, who were excluded. Serum electrolyte levels were determined based on the initial laboratory tests performed upon presentation to the emergency room. The SI was computed by dividing the patient’s blood glucose level (mg/dL) by the potassium level (mEq/L). The research approach entails meticulous documenting of the basic information of all retrieved cases, such as age, sex, previous medical history, injury body area and mechanism, blood-drawn laboratory data, Glasgow Coma Scale (GCS) score [27], Injury Severity Score (ISS) [23,28], and in-hospital mortality.

### 2.2. Statistical Analysis

The chi-square test was used to evaluate categorical variables, and the results are provided in the form of odds ratios (OR) and confidence intervals (CI) with a 95% level of confidence. The presentation of continuous variables is determined by the data distribution, which can be normal or non-normal. Such variables are shown as mean ± standard deviation or median with interquartile range (IQR), respectively. An investigation of whether SI is an independent risk factor for mortality was carried out using logistic regression, which included both univariate and multivariate analyses. Variables were introduced into the multivariate logistic regression model based on their clinical relevance and statistical significance in univariate analyses (*p* < 0.05). To determine the best cutoff value of SI for predicting mortality, the area under the Receiver Operating Characteristic (ROC) curve (AUC) was utilized. This is accomplished by maximizing the sum of the sensitivity (true positive rate) and specificity (true negative rate). Using this method, it is possible to determine the moment at which the SI can differentiate between people who are likely to die and those who are likely to survive. Following this, patients were categorized according to this criterion to ascertain their likelihood of passing away and the adjusted odds ratios (AOR). This classification considers a variety of factors, including age, sex, pre-existing conditions, and injury severity. These variables were selected based on baseline clinical conditions and their known impact on mortality in patients with TBI [28,29,30]. Version 23 of the SPSS Windows software developed by the International Business Machines Corporation (IBM) was used for the statistical analysis. Statistical significance was set at *p* < 0.05.

## 3. Results

### 3.1. Patient Enrollment

**Figure 1:**
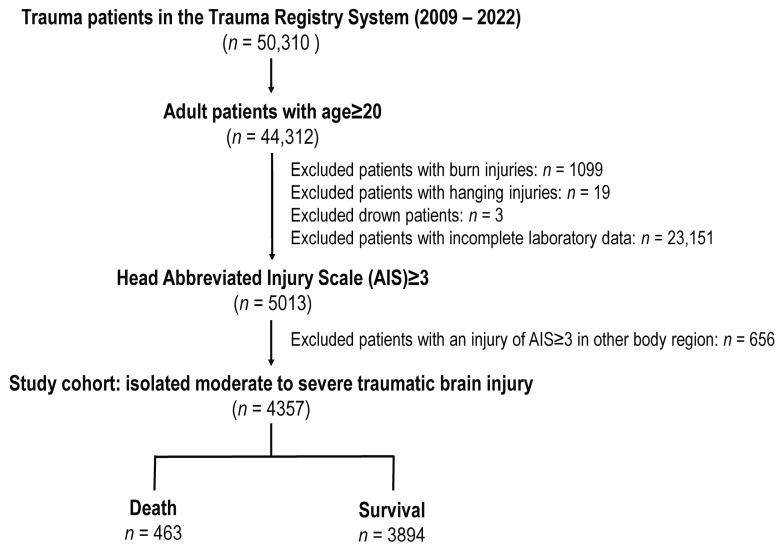
Enrollment of patients into the study cohort.

The study analyzed patients with trauma from the Trauma Registry System spanning 2009 to 2022, focusing on those aged 20 and older with isolated moderate to severe TBI. Initially, the registry contained 50,310 patients. After applying inclusion criteria (age ≥ 20 years), the cohort was reduced to 44,312 patients. Exclusions were then made for patients with burns (*n* = 1099), hanging injuries (*n* = 19), drowning (*n* = 3), and those with incomplete laboratory data (*n* = 23,151), which further narrowed the focus to 5013 patients, in order to identify 4357 patients who had isolated moderate to severe TBI, after the exclusion of 656 patients who had associated injury of AIS ≥ 3 in other body regions. The final cohort included 4357 patients, with 3894 survivors and 463 fatalities (Figure 1).

### 3.2. Patient and Injury Characteristics

Table 1 reveals that majority of the deceased patients were male (67.2 vs. 59.2%, *p* = 0.001), older (average age 62.8 vs. 59.4 years, *p* < 0.001), and had a greater SI (61.7 vs. 44.1, *p* < 0.001). They had considerably higher blood glucose levels (213.2 vs. 158.4 mg/dL, *p* < 0.001) and were more likely to have comorbidities, such coronary artery disease (CAD) and end-stage renal disease (ESRD), than survivors. These patients had significantly lower GCS scores (median (Q1–Q3): 4 (3–9) vs. 15 (11–15), *p* < 0.001), higher ISS (25 (25–29) vs. 16 (16–21), *p* < 0.001), and shorter hospital stays (8.5 vs. 13.3 days, *p* < 0.001).

### 3.3. Univariate and Multivariate Analysis of Mortality Risk Factors

The analysis of mortality risk factors among patients with isolated moderate-to-severe TBI is shown in Table 2. The univariate and multivariate analysis reveals that male sex significantly increases mortality risk (OR 1.41 in univariate and OR 1.70 in multivariate analyses, both *p* < 0.001). Additionally, age was a critical factor, with each additional year increasing mortality risk (OR 1.01; univariate and OR, 1.02; multivariate analyses, both *p* < 0.001). The SI was another significant independent risk factor, with each unit increase escalating the risk of mortality (OR, 1.03; multivariate OR, 6.70; *p* = 0.007). Notably, CAD and ESRD significantly increased the risk of mortality in the univariate analysis (*p* < 0.001 for both), but only ESRD maintained this significance in the multivariate analysis (OR 4.33, *p* < 0.001). We examined the Variance Inflation Factor (VIF) for each predictor variable to assess collinearity among the predictor variables used in the logistic regression models (Table 3). Upon conducting collinearity diagnostics, the VIF values for all predictor variables were found to be well below the general agreement threshold of 5, indicating that collinearity was not a significant concern in our analysis.

### 3.4. The Mortality Predictive Performance of SI

**Figure 2:**
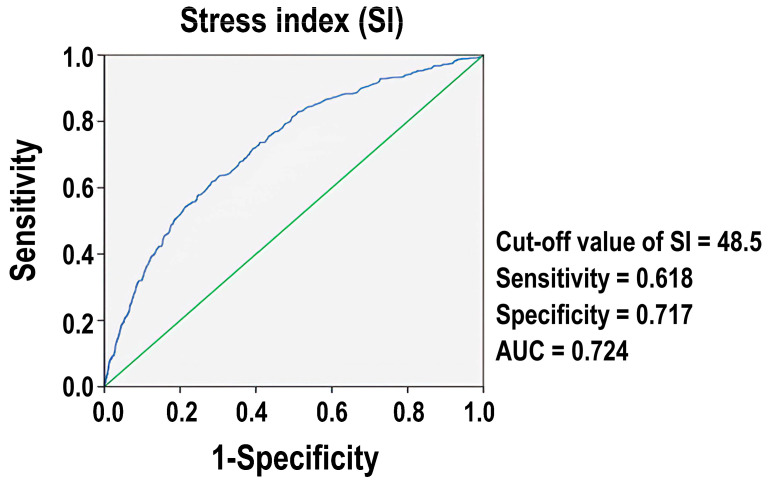
The plot of the Receiver Operating Characteristic (ROC) for the mortality predicting performance of the stress index (blue color). AUC: the area under the Receiver Operating Characteristic curve. The green diagonal line indicates an AUC of 0.5.

Figure 2 presents the performance characteristics of SI as a predictive tool for assessing mortality outcomes. We identified 48.50 as the optimal cut-off value for SI. At this threshold, the sensitivity of the SI to identify patients at risk of death was 62.0%, and the specificity, which measures the ability to correctly identify those not at risk, was 71.4%. The AUC was 0.724, indicating that the SI is a reasonably effective measure with a moderate level of accuracy in predicting mortality outcomes in patients with isolated moderate-to-severe TBI.

### 3.5. Comparative Analysis of the Group of Patients Divided by the Optimal Cut-Off SI Value

Based on the cutoff threshold shown in Table 4, the study compared patients with trauma with an SI of at least 48.5, to those with a lower SI. Significant sex differences were detected, with men having a lower SI and women having a higher SI (men OR 0.81, women OR 1.23; *p* = 0.002). There was no significant difference in age between the two groups. A greater SI was associated with higher rates of hypertension (OR 1.22, *p* = 0.003) and diabetes mellitus (OR 3.16, *p* < 0.001) than a lower SI. Furthermore, these patients presented with worse clinical situations, as reflected in lower GCS scores (median (Q1–Q3): 12 (6–15) vs. 15 (12–15), *p* < 0.001) and higher ISS (20 (16–25) vs.16 (16–20), *p* < 0.001), indicating more severe initial injuries and neurological impairments, which are linked to increased mortality rates. Patients with higher SI had significantly longer hospital stays (15.6 vs. 11.5 days, *p* < 0.001) and higher mortality rates (28.5 vs. 6.0%, *p* < 0.001). The adjusted odds ratio for mortality, controlled by sex, age, comorbidities, GCS, and ISS, was significantly two-fold higher (AOR 1.94, 95% CI: 1.51–2.50, *p* < 0.001).

## 4. Discussion

The results of this study are in accordance with the concept that SI serves as a composite marker that reflects the severity of metabolic disturbances and the extent of cellular injury in response to stress.

Although the exact mechanisms underlying the relationship between elevated SI and adverse TBI outcomes remain to be fully elucidated, the physiological stress response and associated metabolic dysregulation are believed to play a pivotal role. As described by Marini et al. [14], TBI triggers a cascade of cellular events, including the release of neurotransmitters, disruption of membrane potentials, and subsequent ionic imbalances. This cascade ultimately leads to cerebral edema, excitotoxicity, and cellular necrosis, all of which contribute to secondary injury. The SI, which combines serum glucose and potassium levels, can be used to assess the severity of metabolic irregularities and the extent of cellular damage following TBI. Our results further validate the SI as a valuable prognostic indicator, with higher values significantly associated with increased mortality risk even after adjusting for other variables in the analysis. Similar results had been reported that, in patients with severe traumatic brain injury, the SI could predict 30-day mortality [31] and the SI at the time of hospital admission was significantly correlated with the severity of head injuries and was a predictor of poor outcomes, including death or vegetative state during hospitalization [16]. For injuries to other body regions, SI has also been effective in predicting mortality and the need for surgery in patients with isolated blunt thoracoabdominal trauma, surpassing traditional metrics like the shock index [32]. Furthermore, in a study of patients with blunt abdominal trauma, the glucose-to-potassium ratio showed high sensitivity and specificity in predicting mortality [33].

An SI cutoff value of 48.50 was determined in our study, predicting mortality with 62.0% sensitivity, 71.4% specificity, and an AUC of 0.724. This moderate accuracy aligns with the findings of Jung et al. [12] with a cutoff value of 37.8, AUC of 0.747, sensitivity of 90.2%, and specificity of 51.0%, indicating its moderate predictive capability for 3-month mortality in aneurysmal subarachnoid hemorrhage. Similarly, Zhou et al. [31] demonstrated an AUC of 0.777 for the SI in predicting 30-day mortality in patients with severe TBI, reinforcing its robustness. Moreover, Turan et al. [32] reported a higher AUC of 0.854 for the SI in predicting mortality in patients with thoracoabdominal blunt trauma, underscoring its superior predictive role compared with the shock index. SI also demonstrated diagnostic value in differentiating massive from non-massive pulmonary embolisms, with an AUC cutoff value of 26.5, for which the AUC showed 0.733, a sensitivity of 72%, and a specificity of 70% [11]. Collectively, these studies suggest that the SI is a critical indicator for assessing trauma severity and guiding clinical interventions to improve patient outcomes. These findings also suggest that the SI is a valuable biomarker for the early identification and management of high-risk patients, thereby enhancing clinical outcomes through timely and targeted interventions.

Moreover, our findings are consistent with those reported by Shibata et al. [16], who evaluated the prognostic utility of SI for severe TBI. Similar to our results, they observed a significant correlation between poor TBI outcomes (GCS scores of 1 or 2) and elevated SI upon hospital admission. Notably, they reported an OR of 4.079 for a SI ≥ 50, underscoring the potential prognostic value of this biomarker. Our study yielded a similar OR (4.07 for SI > 48.5, in predicting mortality among patients with TBI. Furthermore, our findings align with those of Marini et al. [14], who identified SI as the only biomarker significantly associated with poor outcomes and increased mortality in patients with TBI. Interestingly, Marini et al. [4] reported an OR of 8.61 for a SI > 50, surpassing the OR observed in our study. This discrepancy may be attributed to differences in study populations, distribution of TBI severity, or various types of TBI. Notably, high SI values were not only correlated with patients with TBI but also those with ischemic stroke. A study analyzing patients with ischemic stroke found that those in the highest tertile of SI had an OR of 2.15 for 30-day mortality compared with those in the lowest tertile [13].

Consequently, the SI has emerged as a useful predictive tool for trauma patients with isolated moderate-to-severe TBI. The incorporation of simple but effective biochemical markers, such as SI, into conventional assessment methods for patients with TBI may aid in the early identification of individuals at risk, allowing for focused and potentially life-saving therapies. It can be instrumental in the early identification of high-risk patients with TBI, often correlating with significant physiological stress responses [4,34], which may further reflect increased levels of pro-inflammatory cytokines and a more severe inflammatory response [35]. Although inflammation is a prominent feature of TBI, the point at which it becomes maladaptive and contributes to secondary injury rather than facilitating repair remains unclear [36]. Nevertheless, this holistic approach enables better-informed clinical decisions and potentially improves patient outcomes by tailoring interventions to an individual’s specific physiological state.

Despite these promising findings, retrospective and single-center studies have drawbacks, including potential selection biases, confounding factors, and limited generalizability. Furthermore, patients who were declared deceased at the accident site or upon reaching the emergency department were excluded from the Trauma Registry System. This omission may have led to a biased assessment of the mortality outcomes. The exclusion of a large number of patients without available laboratory data on sugar and potassium levels may also have led to a selection bias. Third, the outcomes of patients may have been affected by the varying approaches of the different surgeons. However, owing to the lack of standardized procedures and clear guidelines for managing diverse clinical circumstances, the reliability of such assessments is questionable. Therefore, we can only assume that the effectiveness of the diagnostic procedures and therapy was consistent among the patients included in this study. The dynamic change in SI over distinct detection time points should also be considered when determining its value. Furthermore, the differences in appropriate SI cut-off values between studies emphasize the importance of standardization and validation in a variety of clinical contexts and patient demographics. To address these limitations and strengthen the evidence supporting SI in TBI management, prospective multicenter studies with larger sample sizes are needed to validate the SI’s predictive performance, establish broadly applicable cutoff values, elucidate the underlying mechanisms, and evaluate the potential utility of SI in guiding therapeutic interventions and clinical decision-making. Understanding the mechanics underlying SI may also help expand its use in clinical settings.

## Figures and Tables

**Table 1:** Patient demographics.

Variables	Death *n* = 463	Survival *n* = 3894	OR (95%CI)	*p*
Sex				0.001 *
Male, *n* (%)	311 (67.2)	2307 (59.2)	1.41 (1.15–1.73)	
Female, *n* (%)	152 (32.8)	1587 (40.8)	0.71 (0.58–0.87)	
Age, years	62.8 ± 18.8	59.4 ± 18.9	-	<0.001 *
Stress index	61.7 ± 30.6	44.1 ± 19.0	-	<0.001 *
Glucose (mg/dL)	213.2 ± 95.7	158.4 ± 63.7	-	<0.001 *
Potassium (mEq/L)	3.6 ± 0.7	3.7 ± 2.3	-	0.225
Comorbidities				
CVA, *n* (%)	23 (5.0)	236 (6.1)	0.81 (0.52–1.26)	0.347
HTN, *n* (%)	181 (39.1)	1508 (38.7)	1.02 (0.83–1.24)	0.878
CAD, *n* (%)	54 (11.7)	282 (7.2)	1.69 (1.24–2.30)	0.001 *
CHF, *n* (%)	2 (0.4)	29 (0.7)	0.58 (0.14–2.43)	0.449
DM, *n* (%)	95 (20.5)	823 (21.1)	0.96 (0.76–1.22)	0.758
ESRD, *n* (%)	36 (7.8)	105 (2.7)	3.04 (2.06–4.50)	<0.001 *
GCS, median (IQR)	4 (3–9)	15 (11–15)	-	<0.001 *
3–8	333 (71.9)	641 (16.5)	13.00 (10.44–16.19)	<0.001 *
9–12	43 (9.3)	458 (11.8)	0.77 (0.55–1.07)	0.115
13–15	87 (18.8)	2795 (71.8)	0.09 (0.07–0.12)	<0.001 *
ISS, median (IQR)	25 (25–29)	16 (16–21)	-	<0.001 *
1–15	16 (3.5)	863 (22.2)	0.13 (0.08–0.21)	<0.001 *
16–24	88 (19.0)	2298 (59.0)	0.16 (0.13–0.21)	<0.001 *
≥25	359 (77.5)	733 (18.8)	14.89 (11.80–18.78)	<0.001 *
Hospital stay (days)	8.5 ± 11.7	13.3 ± 13.4	-	<0.001 *

CAD = coronary artery disease; CHF = congestive heart failure; CI = confidence interval; CVA = cerebral vascular accident; DM = diabetes mellitus; ESRD = end-stage renal disease; GCS = Glasgow Coma Scale; HTN = hypertension; IQR = interquartile range; ISS = injury severity score; OR = odds ratio. * indicate *p* < 0.05.

**Table 2:** Univariate and multivariate analysis of mortality risk factors in trauma patients with isolated moderate to severe traumatic brain injuries.

Variables	Univariate Analysis			Multivariate Analysis		
	OR	CI	*p*	OR	CI	*p*
Male, yes	1.41	(1.15–1.73)	0.001	1.70	(1.34–2.15)	<0.001 *
Age, year	1.01	(1.00–1.02)	<0.001	1.02	(1.02–1.03)	<0.001 *
Stress index	1.03	(1.02–1.03)	<0.001	6.70	(1.66–26.99)	0.007 *
Glucose(mg/dL)	2.20	(1.96–2.46)	<0.001	1.07	(0.72–1.60)	0.728
Potassium (mEq/L)	0.75	(0.62–0.89)	0.001	0.86	(0.66–1.13)	0.281
CAD, yes	1.69	(1.24–2.30)	0.001	1.26	(0.88–1.80)	0.217
ESRD, yes	3.04	(2.06–4.50)	0.001	4.33	(2.71–6.91)	<0.001 *
ISS	1.14	(1.12–1.15)	<0.001	1.14	(1.12–1.15)	<0.001 *

CAD, coronary artery disease; CI, confidence interval; ESRD, end-stage renal disease; ISS, injury severity score; OR, odds ratio. * indicate *p* < 0.05.

**Table 3:** The Variance Inflation Factor (VIF) for each predictor variable used in the logistic regression models.

Predictor Variables	VIF
Age	1.12
Sex	1.07
SI	1.15
Glucose	1.09
Potassium	1.08
CAD	1.10
ESRD	1.14
ISS	1.20

CAD, coronary artery disease; ESRD, end-stage renal disease; ISS, injury severity score; SI = Stress Index; VIF, variance inflation factor.

**Table 4:** Comparative analysis of the groups of patients divided by the optimal cut-off value of the Stress Index (SI).

	Stress Index (SI)			
Variables	≥48.5 *n* = 1385	<48.5 *n* = 2972	OR (95%CI)	*p*
Sex				0.002 *
Male, *n* (%)	785 (56.7)	1833 (61.7)	0.81 (0.71–0.93)	
Female, *n* (%)	600 (43.3)	1139 (38.3)	1.23 (1.08–1.40)	
Age, years	60.1 ± 17.8	59.6 ± 19.4	-	0.407
Comorbidities				
CVA, *n* (%)	70 (5.1)	189 (6.4)	0.78 (0.59–1.04)	0.090
HTN, *n* (%)	582 (42.0)	1107 (37.2)	1.22 (1.07–1.39)	0.003 *
CAD, *n* (%)	117 (8.4)	219 (7.4)	1.16 (0.92–1.47)	0.214
CHF, *n* (%)	14 (1.0)	17 (0.6)	1.78 (0.87–3.61)	0.109
DM, *n* (%)	485 (35.0)	433 (14.6)	3.16 (2.72–3.67)	<0.001 *
ESRD, *n* (%)	40 (2.9)	101 (3.4)	0.85 (0.58–1.23)	0.375
GCS, median (IQR)	12 (6–15)	15 (12–15)	-	<0.001
3–8	536 (38.7)	438 (14.7)	3.65 (3.15–4.24)	<0.001
9–12	171 (12.3)	330 (11.1)	1.13 (0.93–1.37)	0.231
13–15	678 (49.0)	2204 (74.2)	0.33 (0.29–0.38)	<0.001 *
ISS, median (IQR)	20 (16–25)	16 (16–20)	-	<0.001 *
1–15	183 (13.2)	696 (23.4)	0.50 (0.42–0.59)	<0.001 *
16–24	641 (46.3)	1745 (58.7)	0.61 (0.53–0.69)	<0.001 *
≥25	561 (40.5)	531 (17.9)	3.13 (2.71–3.61)	<0.001 *
Hospital stay (days)	15.6 ± 15.8	11.5 ± 11.7	-	<0.001 *
Mortality, *n* (%)	285 (20.6)	178 (6.0)	4.07 (3.33–4.97)	<0.001 *
AOR of mortality *			1.94 (1.51–2.50)	<0.001 *

CAD = coronary artery disease; CHF = congestive heart failure; CI = confidence interval; CVA = cerebral vascular accident; DM = diabetes mellitus; ESRD = end-stage renal disease; GCS = Glasgow Coma Scale; HTN = hypertension; IQR = interquartile range; ISS = injury severity score; OR = odds ratio. * Mortality adjusted by sex, age, comorbidities, GCS, and ISS. * indicate *p* < 0.05.

## Data Availability

The original contributions presented in the study are included in the article, further inquiries can be directed to the corresponding author.
